# Enhancing Thermal Stability of Perovskite Solar Cells through Thermal Transition and Thin Film Crystallization Engineering of Polymeric Hole Transport Layers

**DOI:** 10.1021/acsenergylett.4c01546

**Published:** 2024-08-22

**Authors:** Sanggyun Kim, Sina Sabury, Carlo A. R. Perini, Tareq Hossain, Augustine O. Yusuf, Xiangyu Xiao, Ruipeng Li, Kenneth R. Graham, John R. Reynolds, Juan-Pablo Correa-Baena

**Affiliations:** † School of Materials Science and Engineering, Georgia Institute of Technology, Atlanta, Georgia 30332, United States; ‡ School of Chemistry and Biochemistry, Center for Organic Photonics and Electronics, Georgia Tech Polymer Network, Georgia Institute of Technology, Atlanta, Georgia 30332, United States; § Department of Chemistry, University of Kentucky, Lexington, Kentucky 40506, United States; ∥ National Synchrotron Light Source II, Brookhaven National Laboratory, Upton, New York 11973, United States

## Abstract

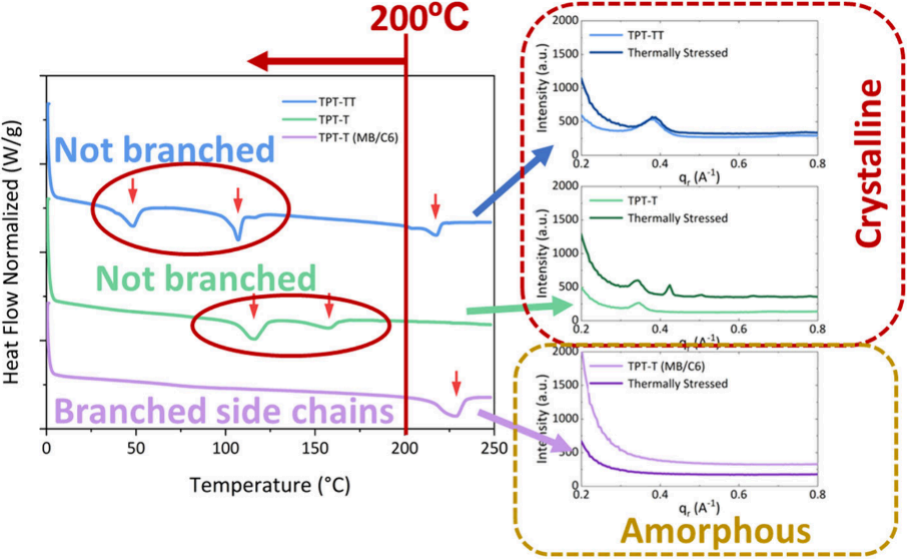

Organic hole transport layers (HTLs) have been known to be susceptible to thermal stress, leading to poor long-term stability in perovskite solar cells (PSCs). We synthesized three 2,5-dialkoxy-substituted, 1,4-bis(2-thienyl)phenylene (TPT)-based conjugated polymers (CPs) linked with thiophene-based (thiophene (T) and thienothiophene (TT)) comonomers and evaluated them as HTLs in n-i-p PSCs. TPT-T (MB/C6), which has branched 2-methylbutyl and linear hexyl (MB/C6) side chains, emerged as a promising HTL candidate, enabling power conversion efficiencies (PCEs) greater than 15%. In addition, PSCs with this HTL showed an improvement in long-term stability at elevated temperatures of 65 °C when compared to those with the state-of-art HTL, 2,2′,7,7′-tetrakis(*N,N-p*-dimethoxyphenylamino)-9,9′-spirobifluorene (spiro-OMeTAD). This improvement is ascribed to the lack of thermal transitions within the operational temperature range of PSCs for TPT-T (MB/C6), which is attributed to the relatively short branched side chains of this polymer. We propose that the elimination of thermal transitions below 200 °C leads to HTLs without cracking as-deposited and after conducting a stress test at 65 °C, which can serve as a new design guideline for HTL development.

Organic–inorganic hybrid perovskite solar cells (PSCs) have made remarkable strides in power conversion efficiency (PCE) over the past decade, reaching an impressive 26.1% for a single junction device.^1^ Despite the high PCE values, long-term operational stability remains a critical challenge for PSC commercialization. Key factors limiting the achievement of long-term stability in devices are the mechanical and chemical changes in the hole transport layer (HTL), which interfaces with the perovskite during operation and thermal cycling.^2^ These solar cells need to withstand elevated temperatures (i.e., 65 °C) in order to pass key International Summit on Organic Photovoltaics Stability (ISOS) metrics.^3,4^ Thus, strategies aimed at enhancing the stability of HTLs in PSCs, while simultaneously maintaining device efficiency, are highly desirable.

Currently, the small molecule 2,2′,7,7′-tetrakis(*N,N-p*-dimethoxyphenylamino)-9,9′-spirobifluorene (spiro-OMeTAD), processed from solution as a thin film, serves as the benchmark HTL. Typically, spiro-OMeTAD is combined with additives such as lithium bis(trifluoromethane)sulfonimide (Li-TFSI) and 4-*tert*-butylpyridine (tBP) to increase the conductivity of the HTL and enable high efficiencies. However, these additives have been shown to migrate toward the HTL interfaces and interact with the perovskite thin film or metal contacts, which leads to long-term stability challenges.^5,6^ In addition to the requirement for additives, the doped spiro-OMeTAD has been shown to crystallize at low temperatures within the range of solar cell operation, leading to the formation of cracks and consequently deteriorating the device performance.^7−10^ To suppress the crystallization of spiro-OMeTAD, researchers have been studying how the molecular engineering of pristine molecules and the incorporation of additives can allow for the control of their thermal transitions.^11^

Conjugated polymer (CP)-based HTLs with improved thermal stability have been investigated as alternatives to spiro-OMeTAD. CPs are attractive HTL candidates owing to their tunable physical and electrical properties, which can be tailored via alteration of the conjugated backbone and side chains.^12^ Poly[bis(4-phenyl)(2,4,6-trimethylphenyl)amine] (PTAA) is the most widely used HTL polymer in PSCs, deposited from solution as a thin film of about 20–50 nm in thickness.^13−15^ PTAA has thermal transitions as low as 98 °C, which is higher than the typical temperatures to which solar cells are exposed during thermal cycling.^16,17^ These thermal transitions are important to the viability of devices, as they are suggested to be responsible for long-term stability under thermal stress. To date, promising thiophene-based CP HTLs have been reported, including poly[3-(4-carboxybutyl)thiophene-2,5-diyl] (P3CT) and poly[3-(6-carboxyhexyl)thiophene-2,5-diyl] (P3HT-COOH).^18,19^ Thiophene-based polymers are some of the most notable systems in CP research, primarily attributed to their electron-rich nature and straightforward synthetic routes.^20,21^ Both P3CT- and P3HT-COOH-incorporated PSCs show encouraging PCEs above 20%. However, there is little understanding of their thermal stability, as stress factors including light, electrical bias, and heat were not introduced simultaneously. For P3CT, thermal stability tests were performed at 85 °C under a N_2_ atmosphere in dark conditions for 144 h without maximum power point tracking (MPPT). The PSC measured over time lost 20% of its original PCE.^18^ Similarly for P3HT-COOH, performance measurements were conducted at 65 °C in a N_2_ atmosphere in dark conditions without MPPT, where the PSC experienced a loss of 20% of its original PCE.

In this work, a family of 1,4-(2-thienyl)-2,5-dialkoxyphenylene (TPT) core units copolymerized with thienothiophene (TT) and thiophene (T) is explored as HTLs in PSCs. These three polymers, with their syntheses presented in Figure S1, are referred to as TPT-TT, TPT-T, and TPT-T (MB/C6). The side chains of the TPT-TT and TPT-T polymers are similar (octyl and decyl pendant from phenyl and the flanking thiophenes), and the π-bridge between the TPT units changes from a fused ring TT to a less electron rich T. In our recent work, TPT-TT has been reported to exhibit a high degree of planarity, promoted by noncovalent intramolecular S–O and S–H–C Coulombic interactions and a high out-of-plane hole mobility of (2.43 ± 0.01) × 10^–4^ cm^2^ V^–1^ s^–1^.^22^ The third polymer (Figure S1), TPT-T (MB/C6), bears a shorter and branched 2-methylbutyl side chain (attached to the phenyl) and linear hexyl side chains (attached to flanking thiophenes). We conducted differential scanning calorimetry (DSC) to probe the physical properties, such as thermal transitions, of the different polymers. The three polymers are deposited as HTL thin films in PSCs and tested under 1 sun conditions.^3^ The PSCs with the TPT-T (MB/C6) polymer as the HTL (without any additives) exhibited a PCE greater than 12%. In addition, we conducted long-term thermal stability measurements following the ISOS protocols.^3^ The TPT-T (MB/C6) shows improved long-term stability compared to TPT-TT and TPT-T. Furthermore, TPT-T (MB/C6) combined with Li-TFSI and tBP additives shows a higher PCE of over 15% and withstands 200 h at 65 °C without significant changes in efficiency.

## Physical Properties of TPT-Based Conjugated Polymers

**Figure 1:**
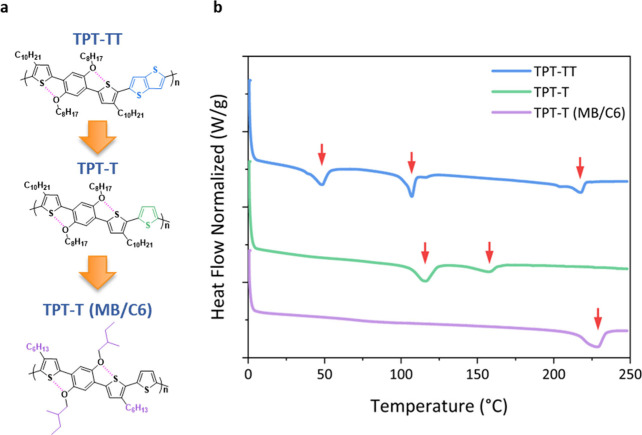
**Molecular structure and DSC curves of newly synthesized thiophene-based conjugated polymer HTLs.** (a) Molecular structures and (b) DSC scans of TPT-TT, TPT-T, and TPT-T (MB/C6). The colored portion of each molecular structure indicates where modifications are made to improve the thermal stability of each conjugated polymer. The red arrows on the DSC data represent different thermal transition points. The heating rate was 10 °C/min in N_2_ atmosphere.

The molecular structures of TPT-TT, TPT-T, and TPT-T (MB/C6) are shown in Figure 1a and are arranged in the sequence of their respective modifications (the synthesis route is shown in Figure S1). The number-average molecular weights (*M*_n_) and dispersity (*Đ*) of TPT-TT, TPT-T, and TPT-T (MB/C6) are 15 kg/mol (*Đ* 1.64), 24 kg/mol (*Đ* 1.51), and 26 kg/mol (*Đ* 2.30), respectively. *M*_n_ and *Đ* values of the polymers were determined by high-temperature gel permeation chromatography (GPC) using 1,2,4-trichlorobenzene at 140 °C as the eluent. GPC traces are shown in Figure S2 and are monomodal. The comparable *M*_n_ and *Đ* values of TPT-T and TPT-T (MB/C6) suggest that any divergences between them are likely attributed to alterations in the backbone and side chain chemistry. The polymer purity was confirmed by elemental analysis and polymer structure using nuclear magnetic resonance (NMR) spectroscopy (Table S1, Figures S3–S5). The detailed synthetic routes of the monomers and Stille cross-coupling polymerization to obtain TPT-based polymers are provided in the Supporting Information (SI).

Examining the repeat unit structures in Figure 1, it can be seen that TPT-TT and TPT-T were designed and synthesized with linear octyloxy side chains on the phenylene unit and linear decyl groups on the thiophene unit. These conformationally flexible side chains provide increased solubility of the polymers for solution processing and, in this instance, lead to multiple thermal transitions, as evident from the DSC results (analyzed from the second heating scan to eliminate thermal history effects) shown in Figure 1b. These types of transitions are expected when a polymer passes through liquid crystalline phases and, as will be discussed later, negatively affect its utility as an HTL. Distinct melting and crystallization features were present in all three polymers, confirming their semicrystalline nature (Figure S6). TPT-TT revealed three thermal transitions at 48 °C, 107 °C, and 218 °C, where the lowest and the highest thermal transitions are due to the side chain and backbone order–disorder, while the transition at 107 °C is correlated with a liquid crystalline behavior.^22^ Replacing a TT with T moves the first thermal transition (side chain order–disorder transition) to 116 °C (from 48 °C in TPT-TT), while the backbone melting drops to 157 °C (compared to 218 °C). These changes are in line with the improvement of the side chain packing strength (higher side chain melting point), while the backbone rigidity and π–π interactions might decrease due to modulation in intramolecular noncovalent interactions and removal of fused ring units from the repeat unit structure.

By shortening and using branched side chains in TPT-T (MB/C6), the overall conformational entropy brought by the side chains is reduced. While this is expected to reduce the solubility of the polymer, we find the solubility to be sufficient for processing useful HTL films. Turning to the DSC, the thermal transition associated with side chain melting was eliminated, and the backbone melting temperature is pushed to temperatures above 200 °C. This relatively high single thermal transition at 228 °C for TPT-T (MB/C6) is indicative of enhanced thermochemical stability at temperatures up to near 200 °C. In general, smaller backbone and side chain structures of conjugated polymers exhibit fewer thermal changes due to more compact packing of polymer chains, which enhances intermolecular interactions and reduces molecular motion.^23−25^

## Optoelectronic Properties

**Figure 2:**
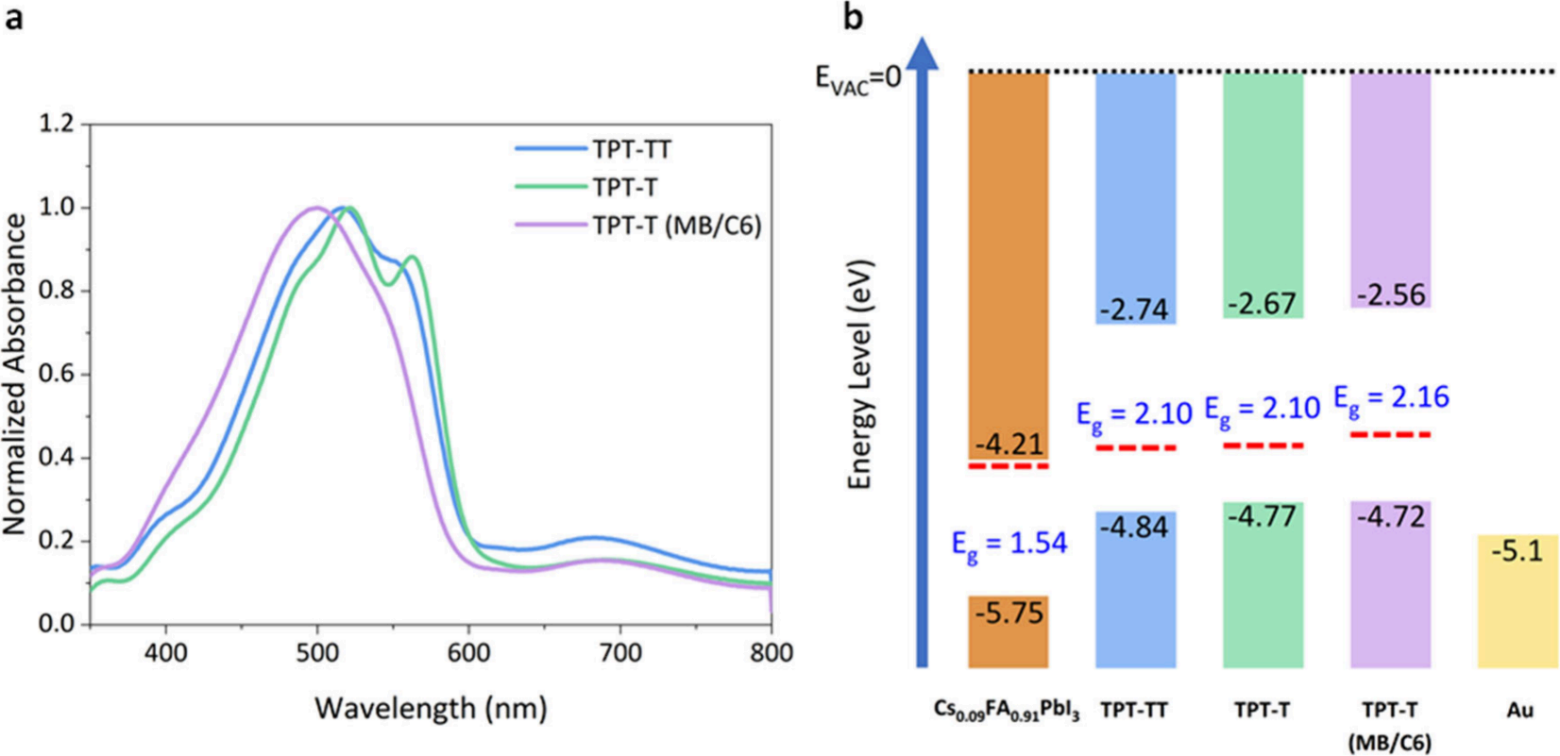
**Optoelectronic properties of TPT-based conjugated polymers.** (a) Normalized UV–vis absorption spectra of TPT-TT, TPT-T, and TPT-T (MB/C6) as thin films on FTO substates. (b) Energy level schematic of the halide perovskite, the TPT-based polymers, and the metal contact. The red dashed line represents the Fermi level energy of each material.

The normalized UV–vis absorption spectra of TPT-TT, TPT-T, and TPT-T (MB/C6) in thin films cast from chlorobenzene at a concentration of 20 mg mL^–1^ via spin coating are shown in Figure 2a. From TPT-TT to TPT-T, the absorption maximum is slightly red-shifted, with the formation of a distinct shoulder peak at 562 nm. TPT-T (MB/C6) shows a blue shift, without any noticeable shoulder peak, when compared to both TPT-T and TPT-TT. Using the onset absorption wavelength of the polymer films, the optical energy band gap (*E*_g_) was also calculated via Tauc’s relationship (Figure S7). The *E*_g_ for both TPT-TT and TPT-T was calculated to be around 2.10 eV, whereas that of TPT-T (MB/C6) was 2.16 eV.

To understand the effects of backbone and side chain modifications in TPT-based polymers on their energetics, we conducted ultraviolet photoelectron spectroscopy (UPS) (Figure S8). The band energy alignments of Cs_0.09_FA_0.91_PbI_3_ (CsFA) perovskite and TPT-based polymers are depicted in Figure 2b (detailed energy positions are shown in Table S2 and Figure S9). TPT-TT shows a HOMO energy (ionization energy) of −4.84 eV. The replacement of thienothiophene with thiophene unit (TPT-T) is accompanied by a slight increase of the HOMO energy level to −4.77 eV. The high energy difference of more than 0.5 eV between the Fermi level and the HOMO level in TPT-based polymers indicates these materials should have relatively low conductivities. Moreover, TPT-T and TPT-T (MB/C6) are anticipated to exhibit electronic properties similar to those of TPT-TT, such as an out-of-plane hole mobility of (2.43 ± 0.01) × 10^–4^ cm^2^ V^–1^ s^–1^, as minimal changes in the Fermi and HOMO levels were observed despite the structural modifications.^22^

## Device Performance of PSCs with TPT-Based Polymers

**Figure 3:**
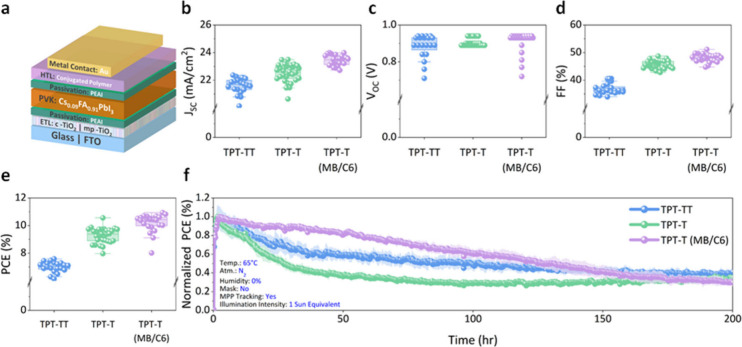
**Photovoltaic performance of TPT-based conjugated polymer HTLs in PSCs.** (a) Device configuration of the n-i-p PSC. (b) *J*_*sc*_, (c) *V*_*oc*_, (d) *FF*, and (e) PCE obtained from reverse *J–V* scans. (f) Long-term stability of PSCs under constant simulated AM 1.5G illumination and MPPT for 200 h with continuous N_2_ flow at 65 °C.

To assess the viability of TPT-based polymers as potential HTLs in PSCs, n-i-p devices consisting of fluorine-doped tin oxide/compact TiO_2_/mesoporous TiO_2_/phenethylammonium iodide (PEAI)/Cs_0.09_FA_0.91_PbI_3_ (CsFA) perovskite/PEAI/CP/Au (Figure 3a) were fabricated. The details of the PSC device fabrication are provided in the SI. The complete device current–voltage characteristics for all devices tested are summarized in Figure S10 and Table S3. Figure 3b–e displays the statistical distributions of short-circuit current density (*J*_*sc*_), open-circuit voltage (*V*_*oc*_), fill factor (*FF*), and power conversion efficiency (PCE) of the devices based on different TPT-based polymer HTLs in reverse scans. Gradual increases in *J*_*sc*_ and *FF* were observed when modifying the polymers from TPT-TT to TPT-T (MB/C6), while *V*_*oc*_ remained relatively constant. The devices with all TPT-based polymers showed an increased series resistance when compared to those prepared using doped Spiro, as inferred from the high-voltage region in the *J–V* curve (Figure S10f). S-shaped *J–V* curves and similar *V*_*oc*_ values of ∼0.94 eV for all TPT-based polymers suggest poor charge extraction between the polymer and Au contact, which could be due to either energy misalignment (Figure 2b) or low hole mobilities leading to charge carrier accumulation.^26^ A PCE of 11.09% (median PCE of 10.04%) was obtained in a PSC based on a TPT-T (MB/C6) HTL. Moreover, a long-term device stability study following the ISOS L-2I protocols was conducted to study the thermal effect on TPT-based polymer-incorporated PSCs, as shown in Figure 3f.^3^ The stability measurements were carried out under constant 1 sun equivalent illumination at 65 °C under a N_2_ atmosphere with constant MPPT. *J–V* scans were performed at 12 h intervals to record the evolution in *J*_*sc*_, *V*_*oc*_, *FF*, and stabilized PCE (Figure S11). Overall, during the 200 h of stress testing, TPT-T (MB/C6) exhibited higher performance and slower decay in stabilized PCE compared to TPT-TT and TPT-T. The improved stability of TPT-T (MB/C6) is primarily due to an unchanged *V*_*oc*_ as time progresses when compared to those of TPT-TT and TPT-T. The *FF* was lower than that of the TPT-TT starting at 108-h mark, while *J*_*sc*_ was similar to that of TPT-TT at the 180-h mark.

## Relation between TPT-Based Polymer Crystallization and PSC Degradation

**Figure 4:**
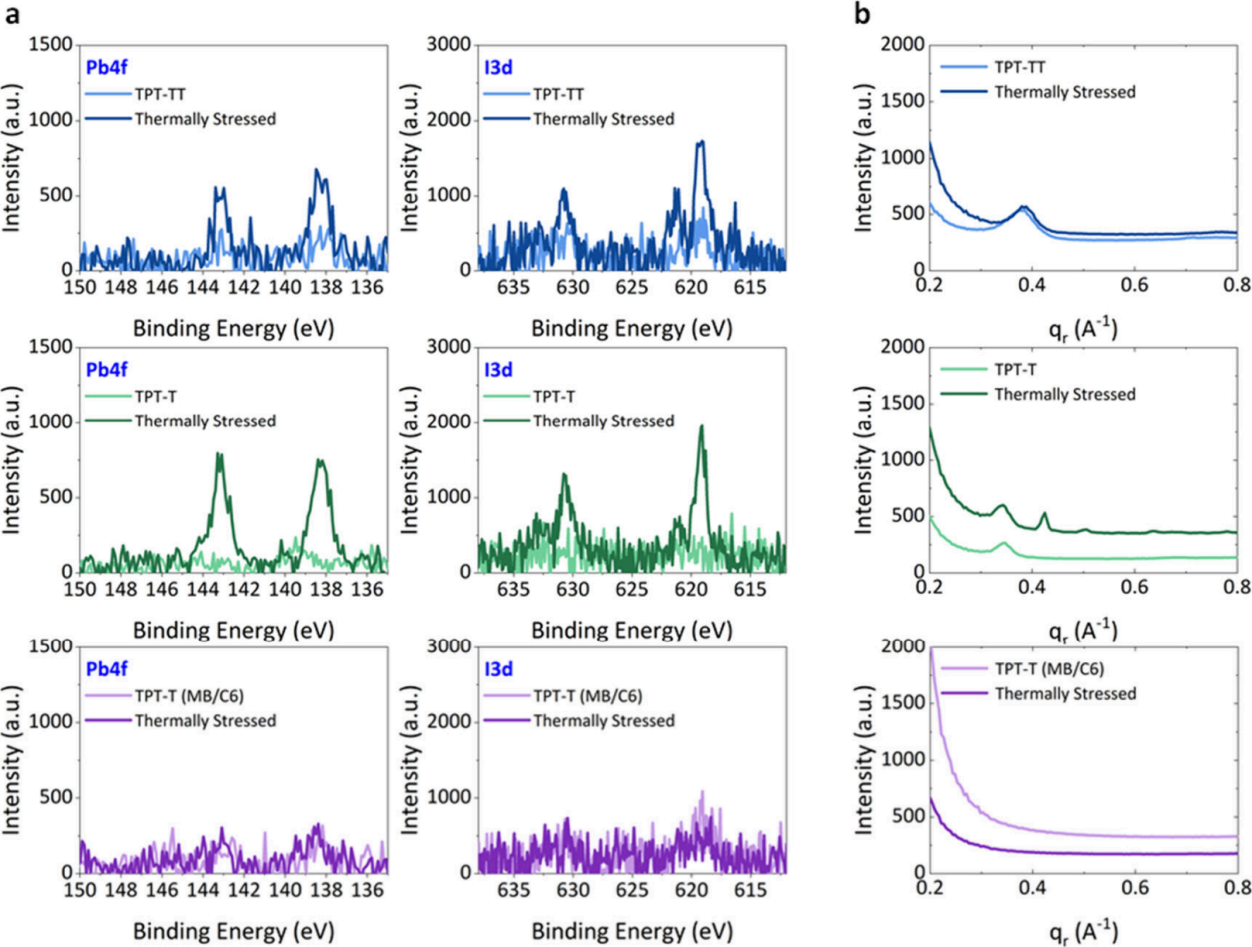
**Chemical composition and structural characterization of PSCs before and after long-term stability test.** (a) Pb 4f and I 3d XPS spectra and (b) 1D integrated GIWAXS patterns on TPT-TT, TPT-T, and TPT-T (MB/C6) surfaces on completed PSCs. GIWAXS data were obtained with a grazing incidence angle of 0.1°.

To investigate the degradation process induced by the ISOS L-2I long-term stability measurement on TPT-based polymer-incorporated PSCs, X-ray photoelectron spectroscopy (XPS) was performed on CPs on top of pristine and aged (for over 200 h at 65 °C) devices. Figure 4a presents the XPS elemental scans of Pb 4f and I 3d of pristine and thermally stressed polymers on the completed PSCs. For all pristine polymers, no significant Pb 4f and I 3d peaks were observed. On the other hand, the Pb 4f and I 3d peaks became more prominent in TPT-TT- and TPT-T-coated films after 200 h of stability test at 65 °C. This suggests that the Pb and I migrate through the polymer to be detected at the surface or that cracks have formed, and we are able to detect those elements through those openings. However, no significant changes were detected for the TPT-T (MB/C6) films, suggesting a lack of elemental migration or crack formation. We conducted scanning electron microscopy (SEM) and optical microscopy (OM) to understand whether the changes in surface chemistry detected by XPS before and after the stability test are associated with microstructural modifications. Figure S12a shows the SEM images of pristine and thermally stressed polymers on the completed PSCs. No signs of crystallization or distinct facets were observed with SEM imaging. However, large features that resemble cracks were observed via OM on both pristine and thermally stressed Spiro, TPT-TT, and TPT-T on completed PSCs (Figure S12b). On the other hand, no crack-like features were observed on TPT-T (MB/C6) films before or after stress testing.

Synchrotron-based grazing incidence wide-angle X-ray scattering (GIWAXS) was performed to assess the effects of long-term stability measurements on the crystallinity of the polymers. Figure 4b shows the 1D integrated GIWAXS profiles of pristine and thermally stressed polymers on completed PSCs. The GIWAXS profiles that include the perovskite signals are shown in Figure S13a. To disentangle the GIWAXS signals of polymers from those of the perovskite layer, we acquired GIWAXS patterns of the polymer thin films deposited on FTO substrates from a 20 mg mL^–1^ solution. The patterns were obtained for films before and after annealing at 100 °C for 20 min in a N_2_ environment to simulate high-temperature aging (Figure S13b). For TPT-TT and TPT-T, crystalline peaks were present for pristine films at *q*_r_ = 0.37 A^–1^ and *q*_r_ = 0.34 A^–1^, respectively. After the thermal stability test, the peaks remained relatively unchanged, while a new peak surfaced for TPT-T at *q*_r_ = 0.42 A^–1^. Importantly, no crystalline peaks were present for the TPT-T (MB/C6)-incorporated PSC before and after the thermal stability test. Since the TPT-T (MB/C6) thin film on FTO substrate showed a small crystalline peak at 0.41 A^–1^, the result suggests that the layer under the polymer also plays an important role in its crystallization.^2^

We previously discussed that the introduction of short branched side chains leads to high thermal transition temperatures in TPT-T (MB/C6) (Figure 1). Our DSC, XPS, OM, GIWAXS, and device stability measurements show a correlation between the temperature at which thermal transitions occur and crystallization in polymer thin films. We believe that the higher temperatures for thermal transitions in TPT-T (MB/C6) are needed to produce a more amorphous as-cast film that does not crystallize at typical operation temperatures of solar cells. The lack of lower-temperature thermal transitions (below 200 °C) is needed for more stable solar cells. On the other hand, the DSC data shows thermal transitions at lower temperatures (below 200 °C) for both TPT-TT and TPT-T, which coincide with crystalline peak formation and cracking for both as-cast and thermally stressed thin films. This, in turn, leads to solar cells that rapidly lose efficiency during thermal stress.

## Effect of Li-TFSI and tBP Additives on TPT-T (MB/C6)

**Figure 5:**
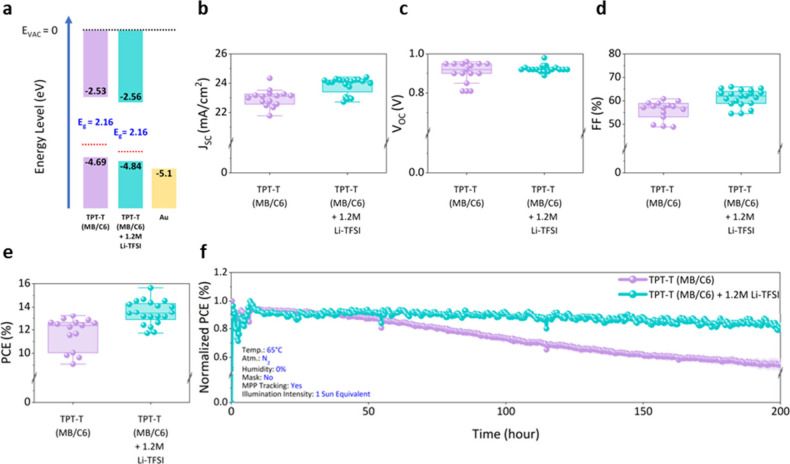
**Photovoltaic performance of PSCs on TPT-T (MB/C6) with Li-TFSI and tBP additives.** (a) Energy level schema of TPT-T (MB/C6) and doped TPT-T (MB/C6). The red dashed lines represent the Fermi level energy of each material. (b) *J*_*sc*_, (c) *V*_*oc*_, (d) *FF*, (e) PCE, and (f) long-term stability of PSCs under constant simulated AM 1.5G illumination and MPPT for 200 h with continuous N_2_ flow at 65 °C. “Li-TFSI” refers to a combination of Li-TFSI and tBP additives.

Having identified TPT-T (MB/C6) as a candidate material for high efficiency and improved long-term stability, we added Li-TFSI with tBP to improve its electronic properties, as is commonly done for such polymeric HTLs. The energy band positions of TPT-T (MB/C6), both with and without the 1.2 M Li-TFSI and tBP additives, are illustrated in Figure 5a (detailed energy positions are shown in Figure S14). The addition of these dopants resulted in a reduction of both the Fermi level and the HOMO energy level, compared to those of the undoped TPT-T (MB/C6). Figure 5b–e shows the statistical distributions of *J*_*sc*_, *V*_*oc*_, *FF*, and PCE of TPT-T (MB/C6) and doped TPT-T (MB/C6) with 1.2 M Li-TFSI and tBP additives; the detailed photovoltaic parameters are reported in Figure S15 and Table S4. Overall improvements in *J*_*sc*_, *V*_*oc*_, and *FF* are observed in the doped TPT-T (MB/C6). The S-shaped *J–V* curve that was originally measured for TPT-T (MB/C6) disappeared for the additive-based devices (Figure S15f), which is attributed to better matching of the HOMO energy level of the HTL to the work function of the Au contact. Furthermore, the lower Fermi level in doped TPT-T (MB/C6) suggests a higher concentration of positive charge carriers, enhancing the conductivity. These improvements led to PCE reaching a maximum of 15.65% for the doped TPT-T (MB/C6). Following the ISOS L-2I protocols, the additive-free and the TPT-T (MB/C6) polymer with additives were subjected to a long-term stability test under 1 sun equivalent illumination at 65 °C in a N_2_ atmosphere with constant MPPT (Figure 5f). A *J–V* scan was conducted every 12 h to track the changes in *J*_*sc*_, *V*_*oc*_, *FF*, and stabilized PCE (Figure S16). Interestingly, the doped TPT-T (MB/C6) did not show much of a change in the PCE for up to 200 h. It is possible that the improved stability of the TPT-T (MB/C6) with additives is due to improved energy level alignment, which is directly related to ion movement in perovskite solar cells.^27^

A series of thiophene-based CP HTLs have been successfully synthesized, characterized, and incorporated into n-i-p PSCs. Among the three CP variants examined, TPT-T (MB/C6) with shorter, branched side chains exhibited superior device performance and exceptional long-term stability. This achievement can be attributed to its amorphous nature, which helps prevent cracking during thermal stress testing. Notably, the TPT-TT and TPT-T polymers were found to undergo faster degradation, marked by the development of crystalline domains and macroscale cracks after thermal stress testing in solar cells. These cracks exposed the underlying perovskite layer. Furthermore, the combination of Li-TFSI and tBP additives with TPT-T (MB/C6) yielded PCE above 15%, which can be attributed to better band alignment and improved electronic properties as the work function increases. Remarkably, TPT-T (MB/C6) with additives exhibited exceptional thermal stability for over 200 h at 65 °C. This work introduces a novel chemical structure design for thiophene-based CPs that not only exhibit thermal resistance but also are compatible with dopants, offering a promising avenue to further enhance the long-term stability of PSCs. Furthermore, our study shows an important correlation between thermal transition temperatures and CP thin film crystallization that occurs within the temperatures at which solar cells are tested. We propose two design rules for organic HTL development: 1) synthesis of HTL materials without thermal transitions below 200 °C and 2) HTL thin films that do not exhibit crystalline peaks before and after thermal stress.

## Supporting Information Available

Details of solution preparation, device fabrication, and characterization (PDF)
