# Supplementary material for: Enhancing Thermal Stability of Perovskite Solar Cells through Thermal Transition and Thin Film Crystallization Engineering of Polymeric Hole Transport Layers
Source: ACS Energy Lett. 2024 Aug 22;9(9):4501–8. doi: 10.1021/acsenergylett.4c01546 (PMC11406513; doi:10.1021/acsenergylett.4c01546)
Supplement: Supplementary file 1 — nz4c01546_si_001.pdf [file nz4c01546_si_001.pdf]

Supplementary Information

Enhancing Thermal Stability of Perovskite Solar Cells through Thermal Transition and Thin Film Crystallization Engineering of Polymeric Hole Transport Layers

Sanggyun Kim [1], Sina Sabury [2], Carlo A.R. Perini [1], Tareq Hossain [3], Augustine O. Yusuf [3], Xiangyu Xiao [1], Ruipeng Li [4], Kenneth R. Graham [3], John R. Reynolds [1,2], Juan-Pablo Correa-Baena [1,2] *

[1] School of Materials Science and Engineering, Georgia Institute of Technology, Atlanta, Georgia 30332, United States.

[2] School of Chemistry and Biochemistry, Center for Organic Photonics and Electronics, Georgia Tech Polymer Network, Georgia Institute of Technology, Atlanta, Georgia, 30332-0400, United States.

[3] Department of Chemistry, University of Kentucky, Lexington, Kentucky 40506, United States

[4] National Synchrotron Light Source II, Brookhaven National Lab, Upton, New York, 11973, United States.

Corresponding author: jpcorrea@gatech.edu

Experimental Methods

<u>TPT-based Conjugated Polymer Materials & Synthesis</u>: The general synthesis scheme for the TPT-based conjugated polymers including the synthesis of the intermediate molecules is shown in **Figure S1**. Side chains are generalized in this figure, labeled as R_1 (side chain attached to the phenyl ring) and R_2 (side chain attached to the flanking thiophene rings). R_1 and R_2 are described for each polymer in **Figure S1**. Details of each synthesis step and the polymerization conditions are reported in our initial report of TPT polymers.[1] The nature of the alkyl side chains (length and linear vs branched) did not affect the synthesis procedure compared to our initial report.

<u>Perovskite Solar Cell Fabrication</u>: Patterned fluorine-doped tin oxide (FTO) glass substrates (7 $\Omega.\ sq^{-1}$) were consecutively cleaned using 2% Mucasol (schülke) solution, deionized water, acetone (Sigma-Aldrich, ≥ 99.5%), and isopropyl alcohol (IPA, Fisher Chemical) via 15 min of ultrasonication. The cleaned substrates were then dried with N_2 gun and put under UV-ozone treatment for 15 min. For an electron transport layer, a compact TiO2 (c-TiO2) layer was deposited by spray pyrolysis from a solution containing 480 μL acetylacetone (Sigma-Aldrich, ≥ 99%), 720 μL titanium diisopropoxide bis(acetylacetonate) 75 wt. % in isopropanol (Sigma-Aldrich), and 10.8 mL of ethanol (Sigma-Aldrich, anhydrous, ≥ 99.5%). The prepared solution was sprayed onto the preheated substrates at 450 °C with 30 s interval between each cycle and then post-annealed for 30 min. A single cycle includes 16 s to 18 s of spraying time, and 3 L min^{-1} flow of O_2 was utilized as a carrier gas. After the substrates were cooled down to room temperature, 60 μL of mesoporous-TiO_2 (mp-TiO_2) solution was static spin coated at a speed of 4000 rpm for 10 s with an acceleration rate of 4000 rpm s^{-1}; mp-TiO_2 solution consists of 150 mg mL^{-1} diluted TiO_2 paste (Sigma-Aldrich) in anhydrous ethanol (Sigma-Aldrich, ≥ 99.5%). As-prepared Glass | FTO | c-

TiO2 | mp-TiO2 stack substrates were desiccated using a hotplate at 100 °C for 10 min and subsequently sintered at 450 °C for 30 min. From cleaning of the substrates to spin coating mp-TiO2 layer was carried out in ambient air and then transferred to N_2 filled glove box.

A 90 μL of phenethyl ammonium iodide (PEAI, Dynamo) solution with a concentration of 1 mg mL^{-1} in IPA (Sigma-Aldrich, 99.9%), was spin coated on top of mp-TiO_2 for 20 s at 5000 rpm with accelerate rate of 5000 rpm s^{-1}. Subsequently, 1.2M $Cs_{0.09}FA_{0.91}PbI_3$ (CsFA) perovskite thin film with 5% excess Pb was deposited using a two-step spin coating process. CsFA solution was prepared by co-dissolving cesium iodide (Sigma-Aldrich), formamidinium iodide (Dynamo), and lead iodide (Tokyo Chemical Industry, > 98.0%) in 2:1 volume ratio of N,N-dimethyl formamide (DMF, Acros Organics, ≥ 99.8%):dimethyl sulfoxide (Acros Organics, 99.8+%). A 90 μL of CsFA perovskite solution was spun at 1000 rpm with acceleration rate of 1000 rpm for 10 s followed by 6000 rpm s^{-1} for 20 s with acceleration rate of 6000 rpm s^{-1}. CsFA perovskite solution was made sure to spread completely over the substrate with a pipette tip before spin coating. Afterwards, 250 uL of chlorobenzene (Sigma-Aldrich, anhydrous, 99.8%) was dynamically spin coated 3 s before the end of the second step, and then annealed at 150 °C for 10 min. The same PEAI surface treatment mentioned above was incorporated on top of the CsFA film and then annealed at 100 °C for 10 min. For the hole transport layer (HTL), a doped Spiro-OMeTAD and 20 mg mL^{-1} of TPT-TT, TPT-T, and TPT-T (MB/C6) in chlorobenzene solution were dynamically spin-cast on the PEAI treated CsFA perovskite film. All of the HTL solutions were prepared at room temperature except for TPT-TT polymer solution which was separately pre-heated at 45 °C for 5 min for better dissolution.

A doped Spiro-OMeTAD consists of 100 mg of Spiro-OMeTAD (1 Material) dissolved in 1098.38 μL of chlorobenzene (Acros Organics, 99.9%) to form a 0.07M solution, 18.13 μL of 1.8M lithium bis(trifluoromethane)sulfonimide (Li-TFSI, Sigma-Aldrich) in acetonitrile (Sigma-Aldrich, anhydrous, 99.8%), 39.45 μL of 4-tertbutylpyridine (tBP, Sigma-Aldrich, 98%) and 9.79 μL of 0.25M tris(2-(1H-pyrazol-1-yl)-4-tert-butylpyridine)cobalt(III) tri[bis(trifluoromethane)sulfonimide] (FK 209 Co (III), Sigma-Aldrich) in acetonitrile; 90 μL of doped Spiro-OMeTAD solution is spin coated dynamically with 3000 rpm for 30 s with an acceleration of 3000 rpm s^{-1}. For TPT- based polymers, 65 μL of polymer solution was dynamically spin coated at a speed of 4000 rpm for 20 seconds with acceleration of 4000 rpm s^{-1}. The solution processing from PEAI treatment to spin coating of hole transport layer was carried out in N_2 filled glove box with O_2 and H_2O content below 4ppm with temperature ranging from 18 °C to 24 °C. The edges of the substrates were cleaned to remove CsFA and hole transport layer with DMF and followed by acetonitrile in ambient air. Finally, 50 nm of Au (Kurt J. Lesker, 99.999%) were thermally evaporated as the back contact using a shadow mask to make 8 independent cells in a single substrate. The active device area is 0.128 cm^2.

<u>Doping of TPT-T (MB/C6)</u>:

Doped TPT-T (MB/C6) was prepared by mixing 20 mg mL^{-1} of TPT-T (MB/C6) with 0.084 mol-to-mol ratio of TPT-T (MB/C6) solution to Li-TFSI (1.2M in acetonitrile) and 0.39 mol-to-mol ratio of TPT-T (MB/C6) to tBP.

Characterization

<u>Elemental Analysis (EA)</u>: EA was conducted via Atlantic Microlab Inc. to calculate polymer repeat units.

<u>Gel Permeation Chromatography (GPC)</u>: The number average molecular weight (M_n), weight average molecular weight (M_w), and dispersity (*Đ*) were determined using a Tosoh EcoSEC high temperature GPC instrument with RI detector.

<u>H^1 Nuclear Magnetic Resonance (NMR)</u>: ^{1}H NMR spectra for all monomers and molecular precursors were acquired through Bruker Avance IIIHD 500 MHz or Bruker Avance IIIHD 700 MHz instruments using $CDCl_3$ as solvent; the residual $CHCl_3$ peak was used as a reference for all reported chemical shifts (^{1}H: δ= 7.26 ppm, ^{13}C: δ= 77.16 ppm).

<u>Differential Scanning Calorimetry (DSC)</u>: DSC was performed using a TA Instruments Q200 with heating and cooling rates of 10 °C min^{-1}. Powder samples with a mass about were used and encapsulated in closed DSC aluminum pan under a controlled N_2 atmosphere.

<u>Ultraviolet-visible (UV-Vis) Spectroscopy</u>: Absorption spectra were measured using Cary 5000 UV-Vis NIR spectrophotometer. The film was prepared by spin-coating 20 mg mL^{-1} polymer solution dissolved in chlorobenzene at room temperature on bare FTO substrate; TPT-TT polymer solution was separately pre-heated at 45 °C for 5 min for better dissolution. The spin coating recipe is dynamic with 65 μL polymer solution at a speed of 4000 rpm for 20 seconds with acceleration of 4000 rpm s^{-1}.

<u>Ultraviolet Photoelectron Spectroscopy (UPS)</u>: UPS was measured inside PHI 5600 UHV chamber coupled with a hemispherical electron energy analyzer. An Excitech H Lyman-α photon source with 10.2 eV photon energy was used. The path of the light was filled with nitrogen. A sample bias of – 5 V and a pass energy of 5.85 eV was used during the measurement. The

secondary electron cut off is determined by linear fitting in both polymer and perovskite samples and is used to calculate the work function by subtracting it from the photon source energy (10.2 eV). The highest molecular orbital (HOMO) onset for the hole transporting layers (HTL) is determined by a linear fitting to the HOMO onset, with the onset energy difference from the Fermi energy added to the work function to obtain the HOMO energy with respect to the vacuum. For the perovskite film, the valence band maximum (VBM) onset is determined by a Gaussian fitting method, as previously reported.[2] The combination of the optical energy band gap from UV-Vis and HOMO/VBM energy extracted from UPS is used to estimate the lowest unoccupied molecular orbital energy level (LUMO)/conduction band minimum (CBM).

All films were spin-coated on top of 0.4M CsFA perovskite thin film with 5% excess Pb on bare tin-doped indium oxide (ITO) substrate and characterized by UPS. A 90 μL of 0.4M CsFA perovskite solution was spun at 1000 rpm with acceleration rate of 1000 rpm for 10 s followed by 6000 rpm s^{-1} for 20 s with acceleration rate of 7000 rpm s^{-1}. Subsequently, 250 uL of chlorobenzene was dynamically spin coated 3 s before the end of the second step, and then annealed at 150 °C for 10 min. For both undoped and doped Spiro-OMeTAD, 0.02M solutions were prepared. A 90 uL of solution is spin coated dynamically with 5000 rpm for 30 s with an acceleration of 5000 rpm s^{-1}. For TPT-based polymers, a 65 uL of 15 mg mL^{-1} polymer solutions were dynamically spin coated at a speed of 6000 rpm for 20 seconds with acceleration of 6000 rpm s^{-1}.

<u>Scanning Electron Microscopy (SEM) & Optical Microscopy (OM)</u>: SEM measurements were carried out with Hitachi SU8230 using a secondary-electron detector at 1.5 keV and 10 mA emission current. Optical microscope images were acquired with LEICA DM 2500 optical microscope.

<u>Grazing Incidence Wide-Angle X-ray Scattering (GIWAXS)</u>: Synchrotron GIWAXS

measurements were performed at beamline 11-BM at National Synchrotron Light Source II in Brookhaven National Laboratory. The X-ray beam energy of 13.5 keV with a spot size of 0.2 mm x 0.05 mm. The samples were irradiated for 10 s with an incident angle of 0.05°, 0.1°, and 0.5°. Beam divergence was 1 mrad with energy resolution of 0.7%. The data were analyzed using the SciAnalysis packaged provided by the beamline.

X-ray Photoelectron Spectroscopy (XPS): XPS measurements were conducted with a Thermo Scientific K-Alpha using a monochromatic Al Kα X-ray source (hv = 1486.6 eV) with a 60° incident angle and a 0° photoemission angle, both measured from the samples normal vector. Survey and high-resolution scans were measured when the chamber pressure was less than $1x10^{-7}$ torr. Survey scans were acquired averaging two measurements with 200 eV pass energy, 50 ms dwell time, and 0.1 eV step size. High-resolution scans were collected averaging 10 measurements with 50 eV pass energy, 50 ms dwell time, and 0.1 eV step size for Au4f, C1s, Cs3d, Co2p, F1s, I3d, N1s, O1s, Pb4f, S2p. Peak fitting was calculated via Thermo Scientific Advantage Data System. For all peaks, C-C peak position was used as a reference binding energies to compensate for possible surface charge shifting.

Device Characterization: Photovoltaic performance was evaluated with Fluxim Litos Lite setup equipped with a Wavelabs Sinus-70 AAA solar similar with an illumination of AM 1.5G at room temperature and in ambient air. The current density-voltage (J-V) characteristics were measured in both forward and reverse scans from 1.2 V to -0.5 V with a scan rate of 50 mV s^{-1}. Max power point tracking (MPPT) algorithm was utilized to ascertain stabilized power output of 120 seconds. Masking was also adopted to define cell area of 0.0625 cm^2 within the active device area of 0.128 cm^2. During the characterization, N_2 gas flow was introduced, and temperature was not regulated.

All devices were measured without pre-conditioning such as light-soaking and applied bias voltage.

For the long-term thermal stability measurement, a Fluxim Litos, a stress-test platform for degradation analysis, was employed. The solar cells were stressed at temperature of 65 °C in N_2 rich environment, under 1 sun equivalent illumination without UV and continuous MPP tracking. This follows the International Summit on Organic PV Stability (ISOS) L-2I protocols for stability measurement; L, 2, and I stand for light on with bias, high temperature, and inert atmosphere, respectively.[3] During the stability measurement, automated acquisition of J-V scans in both reverse and forward directions were taken every 12 hours.

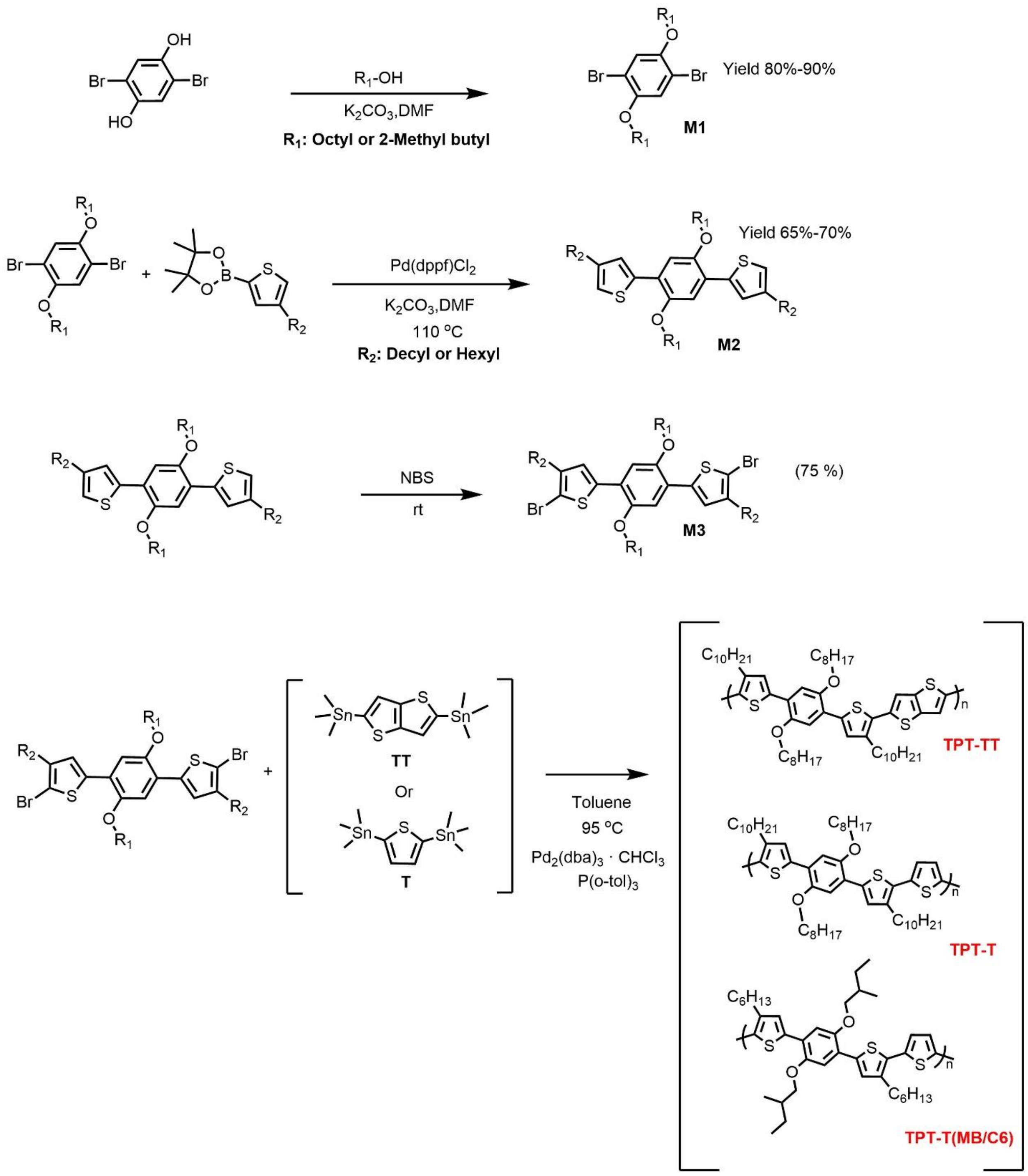

Figure S1. The general synthesis scheme for the three TPT-based conjugated polymers, TPT-TT (R_1: Octyl, R_2: Decyl), TPT-T (R_1: Octyl, R_2: Decyl), and TPT-T(MB/C6) (R_1: 2-Methyl butyl, R_2: Hexyl).

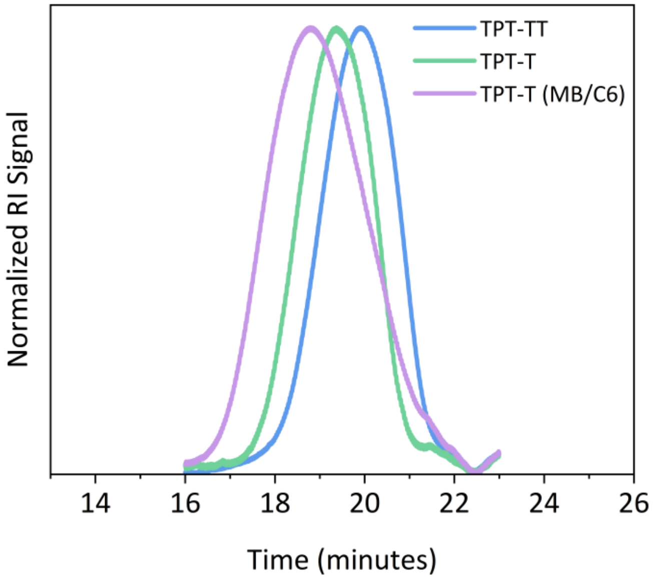

Figure S2. GPC trace of the TPT-based polymers, TPT-TT (Mn = 15 kg/mol, Đ: 1.64), TPT-T (Mn = 24 kg/mol, Đ: 1.51), and TPT-T(MB/C6) (Mn = 26 kg/mol, Đ: 2.3) using 1,2,4-trichlorobenzene as solvent at high temperature (140 °C).

Table S1. Elemental composition analysis of the TPT polymers to ensure purity of the samples.

Polymer	Theoretical composition	Measured Composition
**TPT-TT**	**C**:73.47%, **H**:9.03%, **S**:14.01%	**C**:72.84%, **H**:9.02%, **S**:14.40%
**TPT-T**	**C**:75.47%, **H**:9.62%, **S**:11.19%	**C**:74.77%, **H**:9.58%, **S**:10.94%
**TPT-T(MB/C6)**	**C**:72.46%, **H**:8.21%, **S**:14.51%	**C**:71.89%, **H**:8.07%, **S**:14.25%

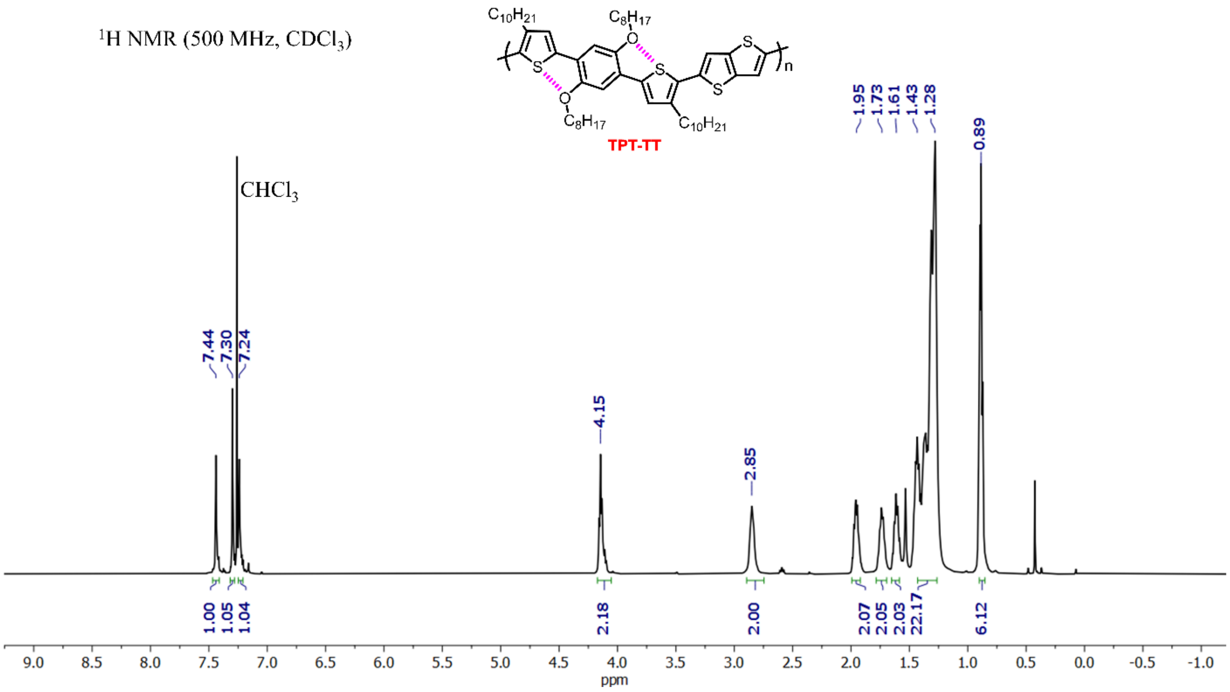

Figure S3. ^{1}H NMR spectrum of TPT-TT (500 MHz, $CDCl_3$, 25 °C). δ(ppm): 7.44 (s, 1H), 7.30 (s, 1H), 7.24 (s, 1H), 4.15 (m, 2H), 2.85 (m, 2H), 1.99-1.90 (m, 2H), 1.78-1.68 (m, 2H), 1.68-1.51 (m, 2H), 1.42-1.26 (m, 22H), 0.90-0.86 (m, 6H).

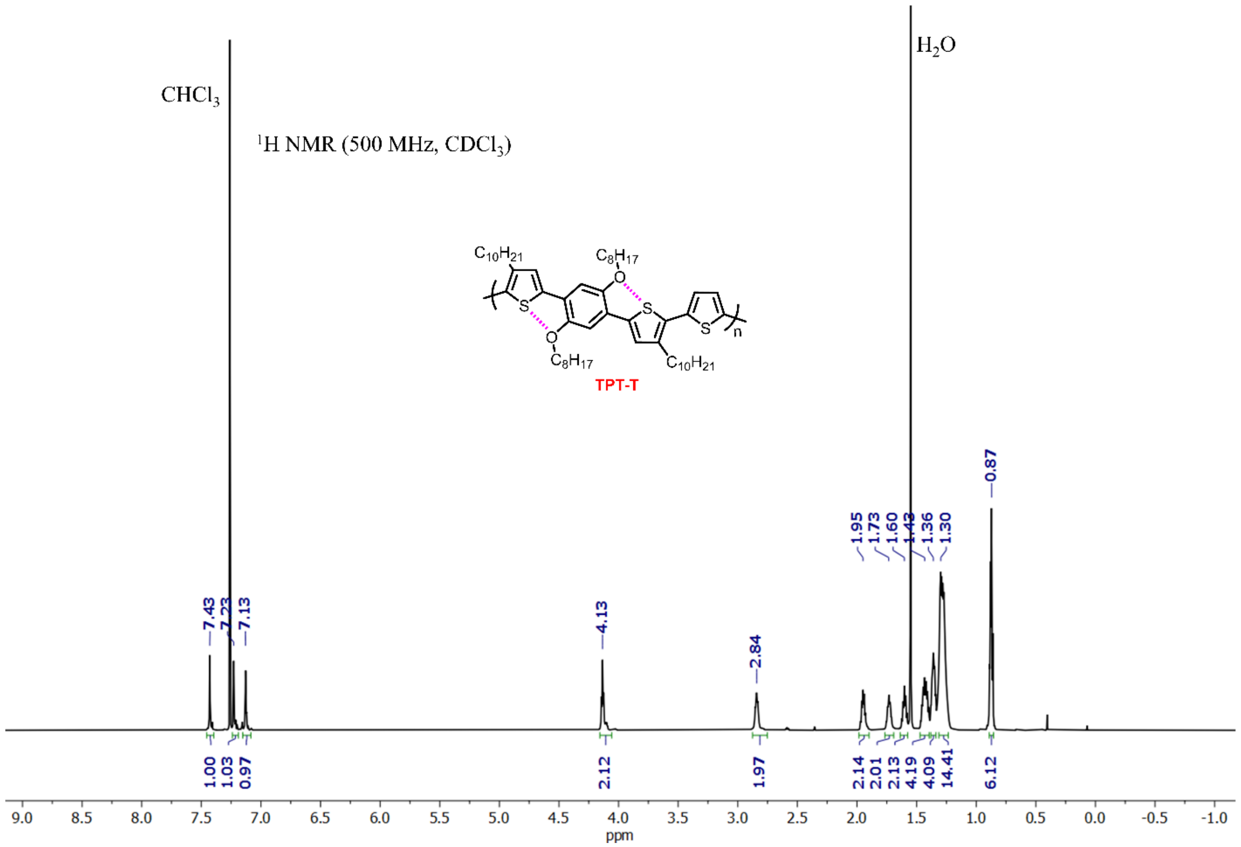

Figure S4. ^{1}H NMR spectrum of TPT-T (500 MHz, $CDCl_3$, 25 °C). δ(ppm): 7.43 (s, 1H), 7.23 (s, 1H), 7.13 (s, 1H), 4.13 (m, 2H), 2.84 (m, 2H), 1.98-1.89 (m, 2H), 1.76-1.79 (m, 2H), 1.63-1.57 (m, 2H), 1.46-1.39 (m, 4H), 1.37-1.34 (m, 4H), 1.31-1.23 (m, 14H), 0.89-0.85 (m, 6H).

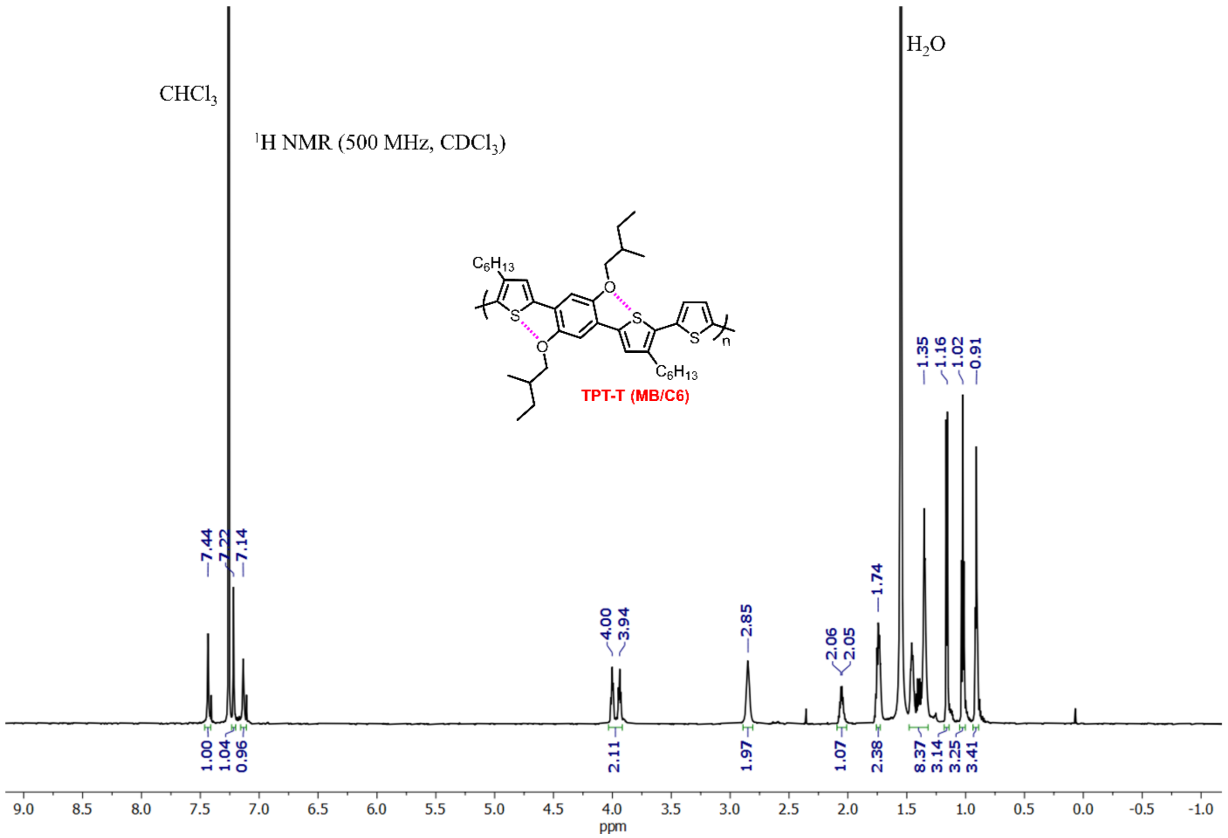

Figure S5. ^1H NMR spectrum of TPT-T (500 MHz, $CDCl_3$, 25 °C). δ(ppm): 7.43 (s, 1H), 7.23 (s, 1H), 7.13 (s, 1H), 4.13 (m, 2H), 2.84 (m, 2H), 1.98-1.89 (m, 2H), 1.76-1.79 (m, 2H), 1.63-1.57 (m, 2H), 1.46-1.39 (m, 4H), 1.37-1.34 (m, 4H), 1.31-1.23 (m, 14H), 0.89-0.85 (m, 6H).

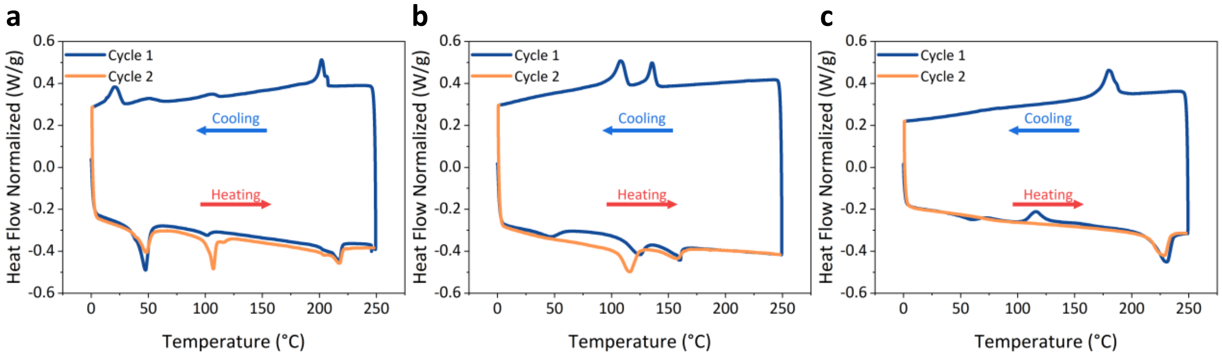

Figure S6. DSC thermograms of (a) TPT-TT, (b) TPT-T, and (c) TPT-T (MB/C6) measured at a scan rate of 10 °C/min in N_2 atmosphere.

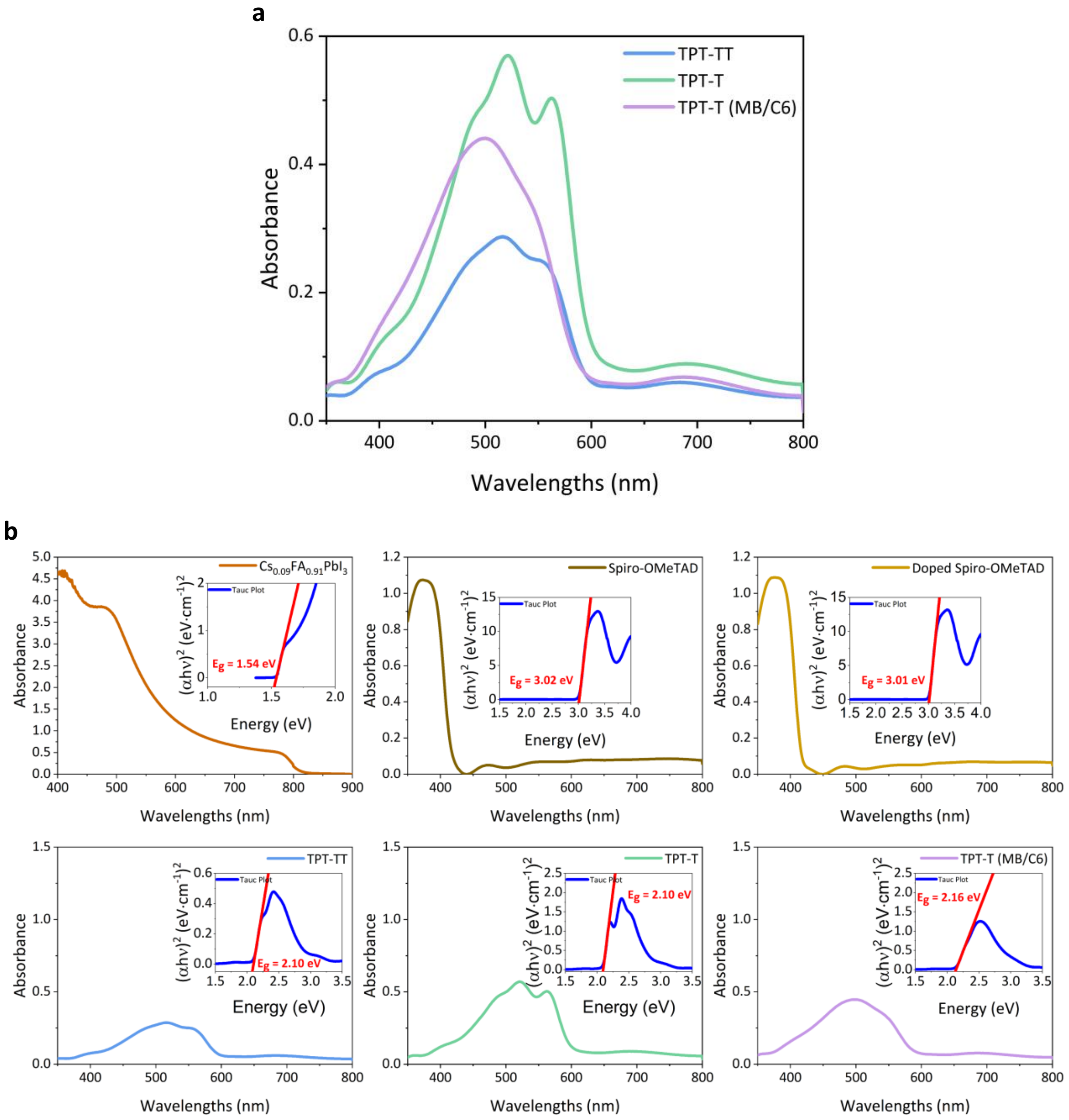

Figure S7. (a) UV-Vis absorption spectra of TPT-based conjugated polymer thin films. (b) Optical energy band gap of CsFA perovskite and HTL thin films used in this work via Tauc method.

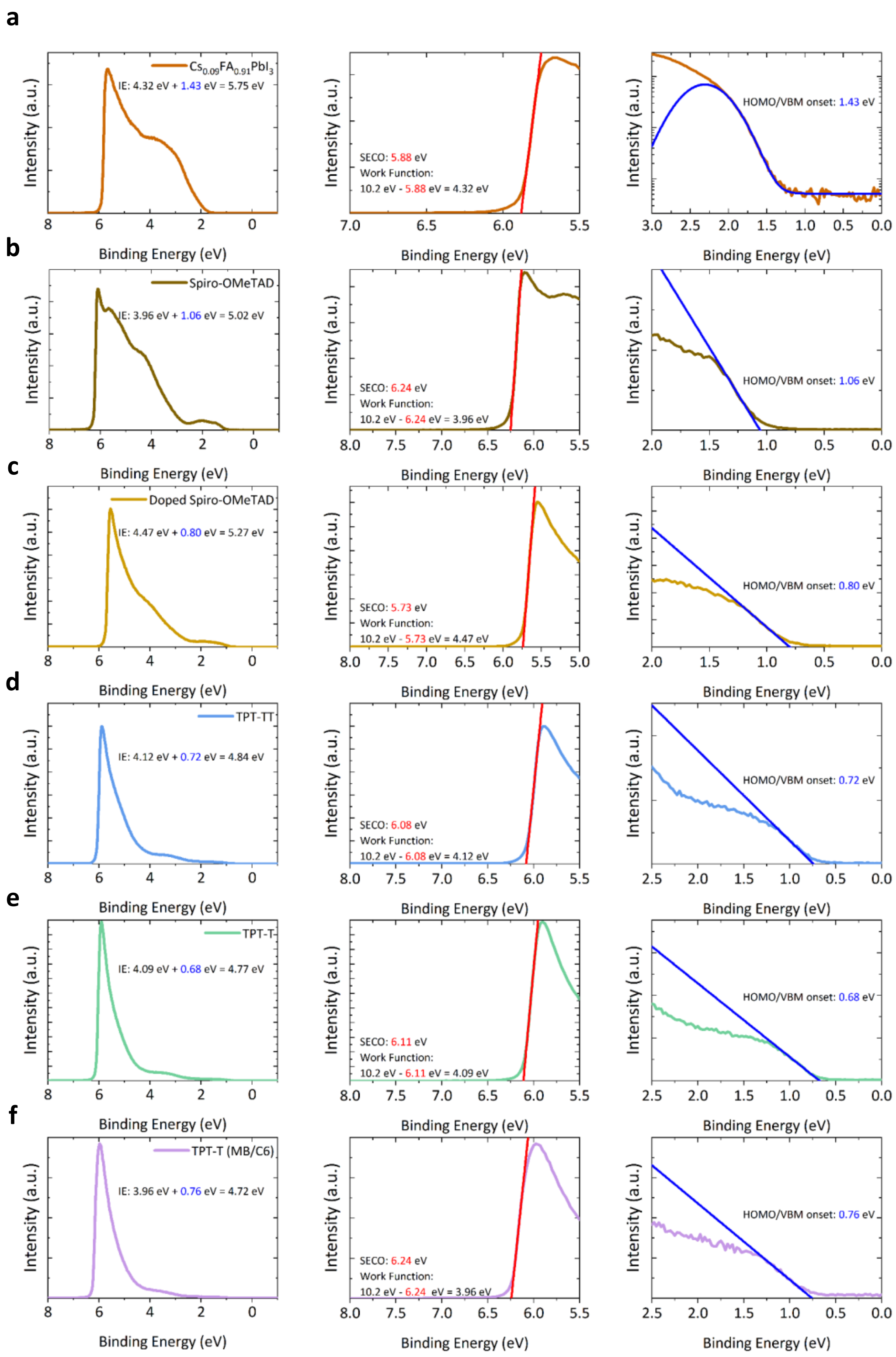

Figure S8. UPS spectrum, secondary electron cut-off (SECO), and valence band onset of (a) CsFA perovskite, (b) Spiro-OMeTAD, (c) Doped Spiro-OMeTAD, (d) TPT-TT, (e) TPT-T, and (f) TPT-T (MB/C6).

Table S2. Optoelectronic properties of CsFA perovskite and HTLs determined by combination of UV-Vis and UPS measurements.

Thin Film	Optical Energy Band Gap (eV)	Work Function (eV)	Valence Band Maximum (eV)	Calculated Conduction Band (eV)
$Cs_{0.09}FA_{0.91}PbI_3$	1.54	4.32	5.75	4.21
Spiro-OMeTAD	3.02	3.96	5.02	2.00
Doped Spiro-OMeTAD	3.01	4.47	5.27	2.26
TPT-TT	2.10	4.12	4.84	2.74
TPT-T	2.10	4.09	4.77	2.67
TPT-T (MB/C6)	2.16	3.96	4.72	2.56

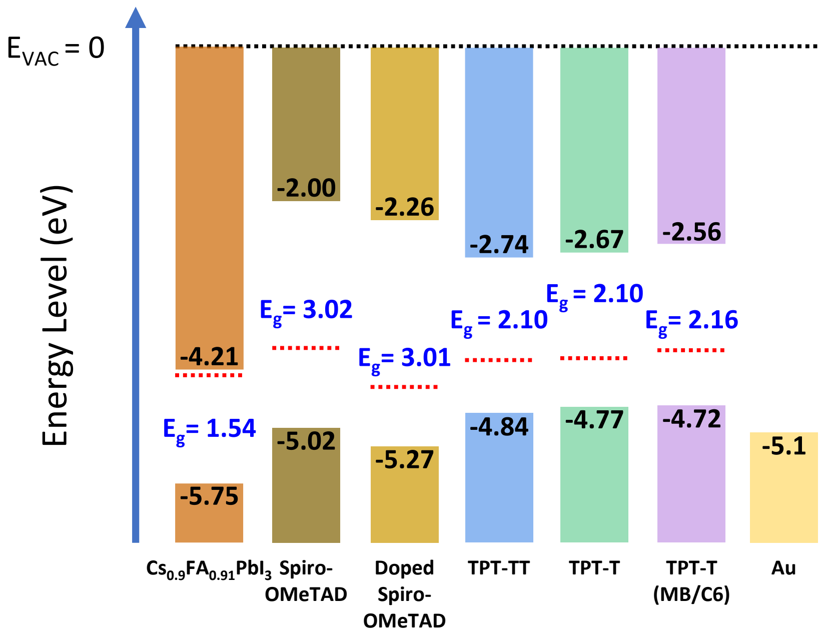

Figure S9. Energy level of CsFA perovskite and different HTL thin films. The red dashed line represents the Fermi level energy of each material.

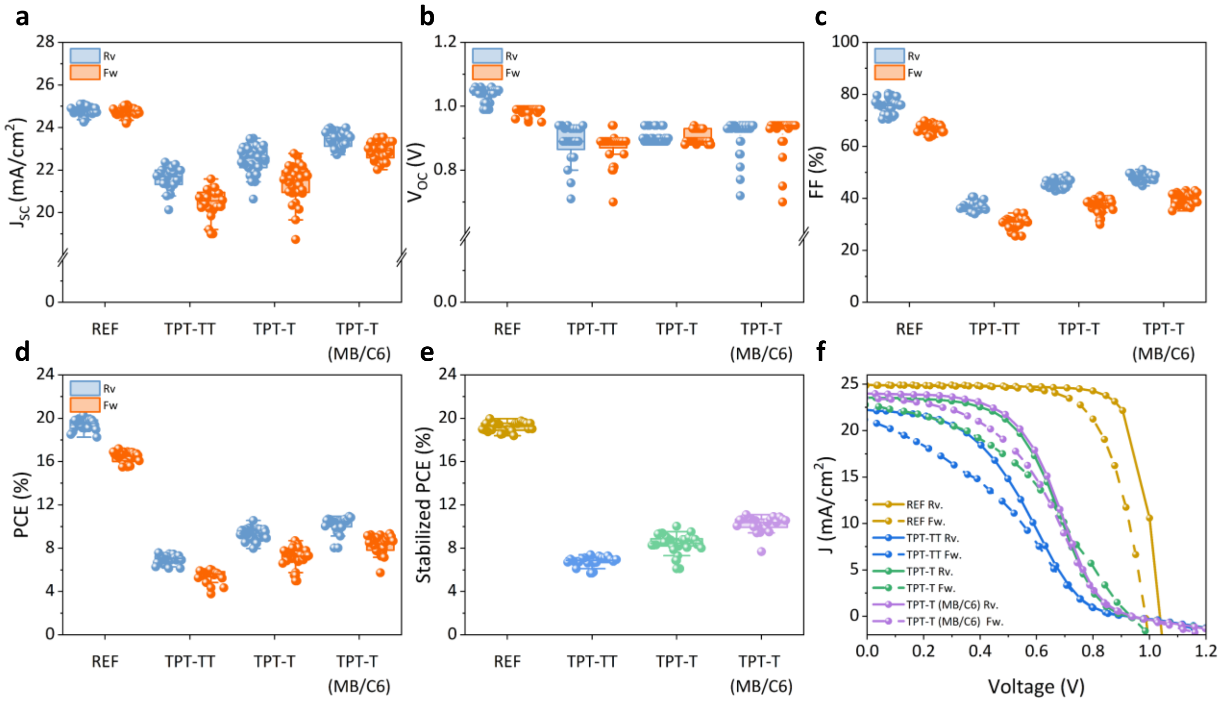

Figure S10. Statistics of (a) Jsc, (b) V_{OC}, (c) FF, (d) PCE, (e) stabilized PCE, and (f) J-V curves of champion devices for REF and TPT-based conjugated polymers incorporated PSCs. REF refers to “doped Spiro-OMeTAD” used for control.

Table S3. Summarized photovoltaic parameters extracted from the reverse J-V curves of PSCs from Fig. S10.

HTL	Max J_{SC} (mA cm^{-2})	Median J_{SC} (mA cm^{-2})	Max V_{OC} (V)	Median V_{OC} (V)	Max FF (%)	Median FF (%)	Max PCE (%)	Median PCE (%)	Max Stabilized PCE (%)	Median Stabilized PCE (%)
**REF**	25.1	24.66	1.06	1.03	81.31	75.96	20.29	19.4	20.15	19.13
**TPT-TT**	22.37	21.68	0.94	0.89	40.61	36.15	7.59	6.96	7.39	6.76
**TPT-T**	23.5	22.6	0.94	0.9	48.71	45.41	10.56	9.38	10.04	8.36
**TPT-T (MB/C6)**	23.99	23.48	0.94	0.93	51.17	48.06	10.94	10.43	11.09	10.04

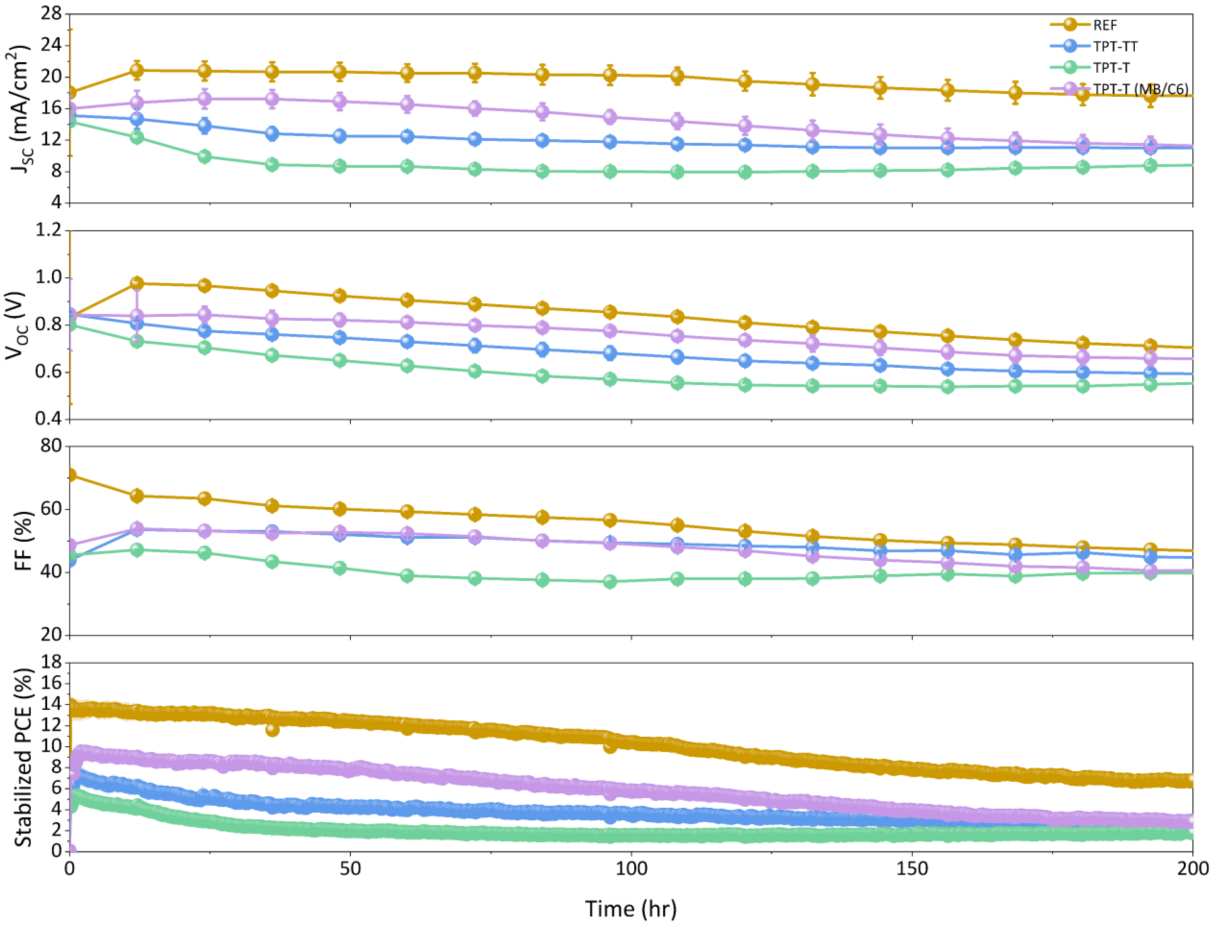

Figure S11. Evolution of the J-V parameters during long-term stability measurement of REF, TPT-TT, TPT-T, and TPT-T (MB/C6). Photovoltaic parameters from J-V scans were automatically extracted every 12 hours during the MPPT at 65 °C. REF refers to "doped Spiro-OMeTAD" used for control.

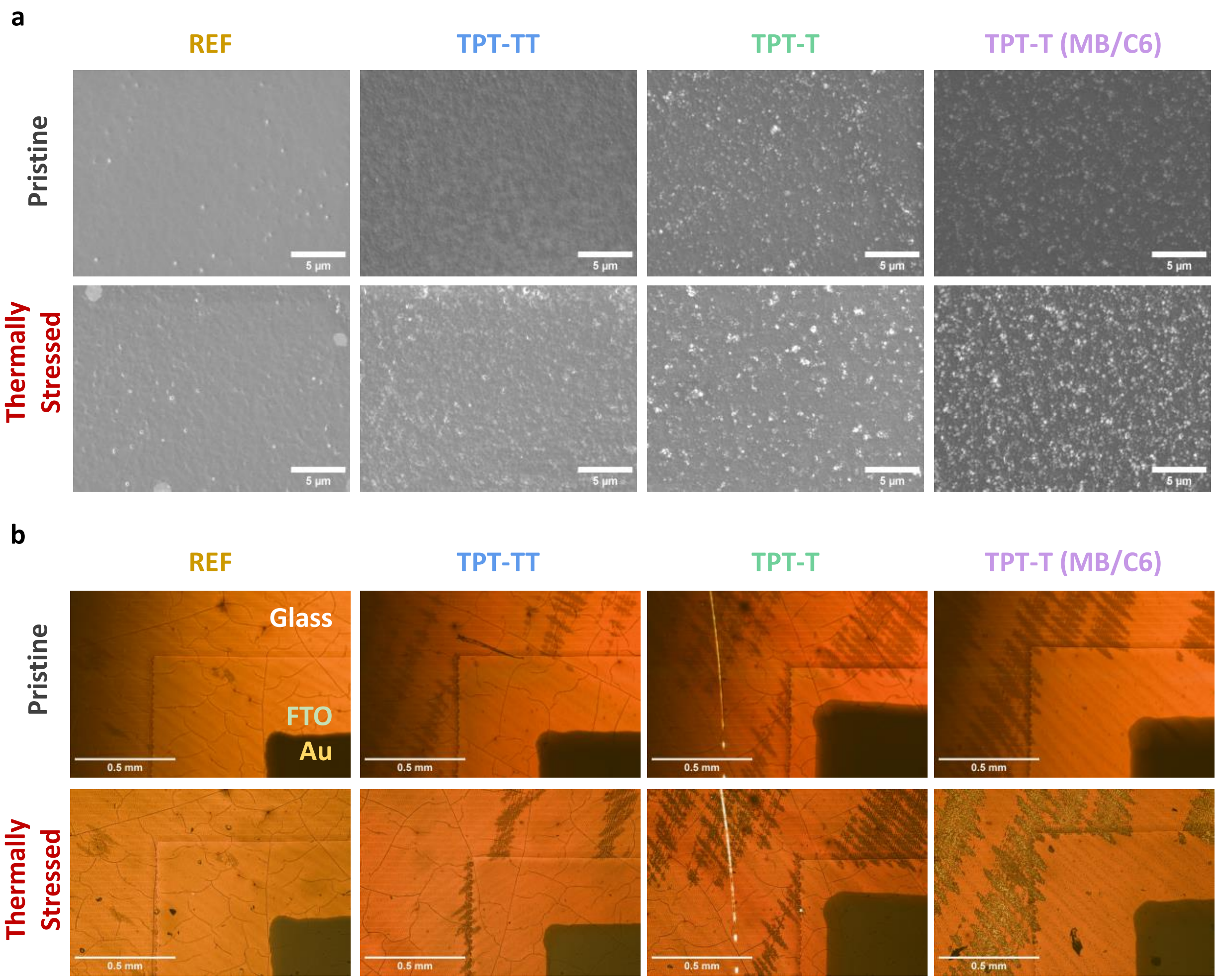

Figure S12. (a) Top-view SEM and (b) OM images of pristine and thermally stressed REF, TPT-TT, TPT-T, and TPT-T (MB/C6) on completed PSCs. For SEM, no obvious change in morphology was found for TPT-based polymers due to their transparency. REF refers to "doped Spiro-OMeTAD" used for control.

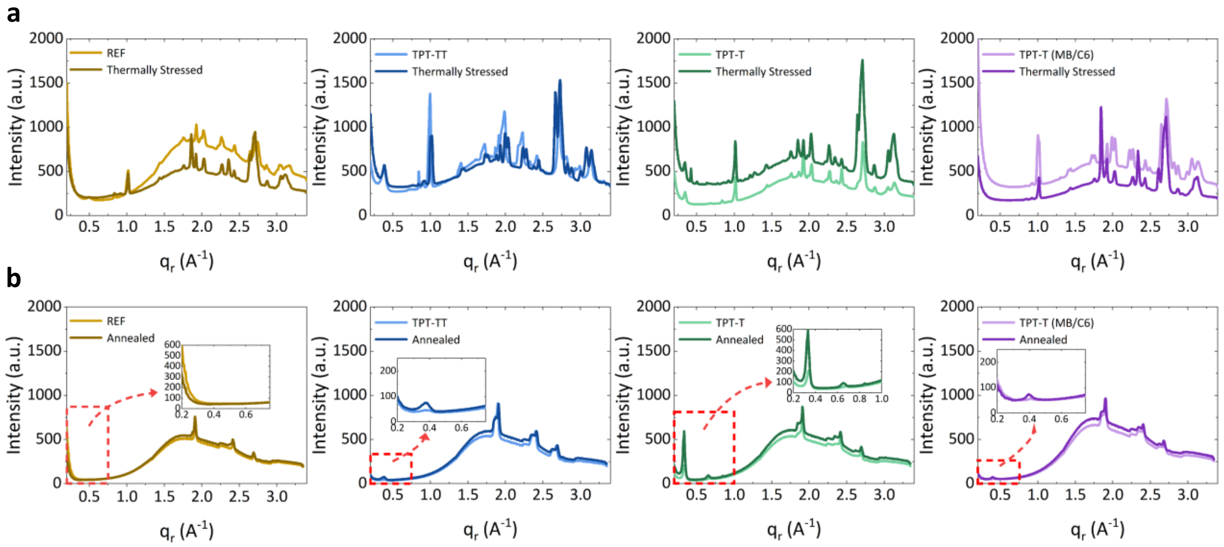

Figure S13. (a) 1D integrated GIWAXS profiles of REF, TPT-TT, TPT-T, and TPT-T (MB/C6) (a) on completed PSCs before and after long-term stability measurement and (b) on FTO substrates before and after annealing at 100 °C for 20 min. REF refers to “doped Spiro-OMeTAD” used for control.

a

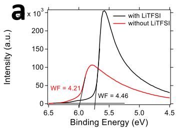

b

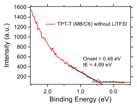

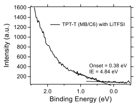

Figure S14. (a) Electron cut-off (SECO) and (b) valence band onset of undoped and doped TPT-T (MB/C6). “Li-TFSI” refers to combination of Li-TFSI and tBP additives.

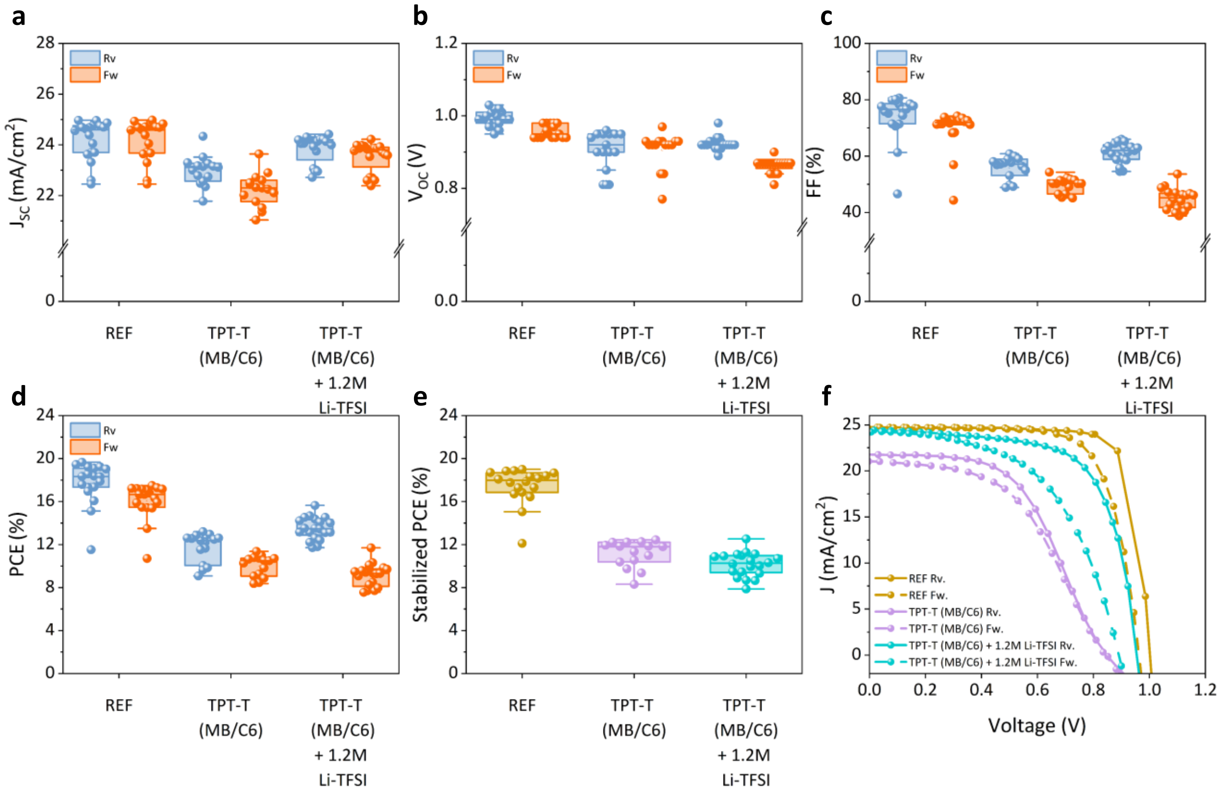

Figure S15. Statistics of (a) Jsc, (b) V_{OC}, (c) FF, (d) PCE, (e) stabilized PCE, and (f) J-V curves of champion devices for REF, TPT-T (MB/C6), and doped TPT-T (MB/C6) incorporated PSCs. REF and 1.2M Li-TFSI refers to "doped Spiro-OMeTAD" used for control and combination of Li-TFSI and tBP additves, respectively.

Table S4. Summarized photovoltaic parameters extracted from the reverse J-V curves of PSCs from Fig. S15.

HTL	Max J_{SC} (mA cm^{-2})	Median J_{SC} (mA cm^{-2})	Max V_{OC} (V)	Median V_{OC} (V)	Max FF (%)	Median FF (%)	Max PCE (%)	Median PCE (%)	Max Stabilized PCE (%)	Median Stabilized PCE (%)
**REF**	24.97	24.6	1.03	0.99	80.65	76.56	19.65	18.32	19.00	17.31
**TPT-T (MB/C6)**	24.34	23	0.96	0.9	60.87	55.01	13.21	11.54	12.44	10.06
**TPT-T (MB/C6) + 1.2M Li-TFSI**	24.42	24.06	0.98	0.92	65.96	61.99	15.65	13.45	12.53	8.36

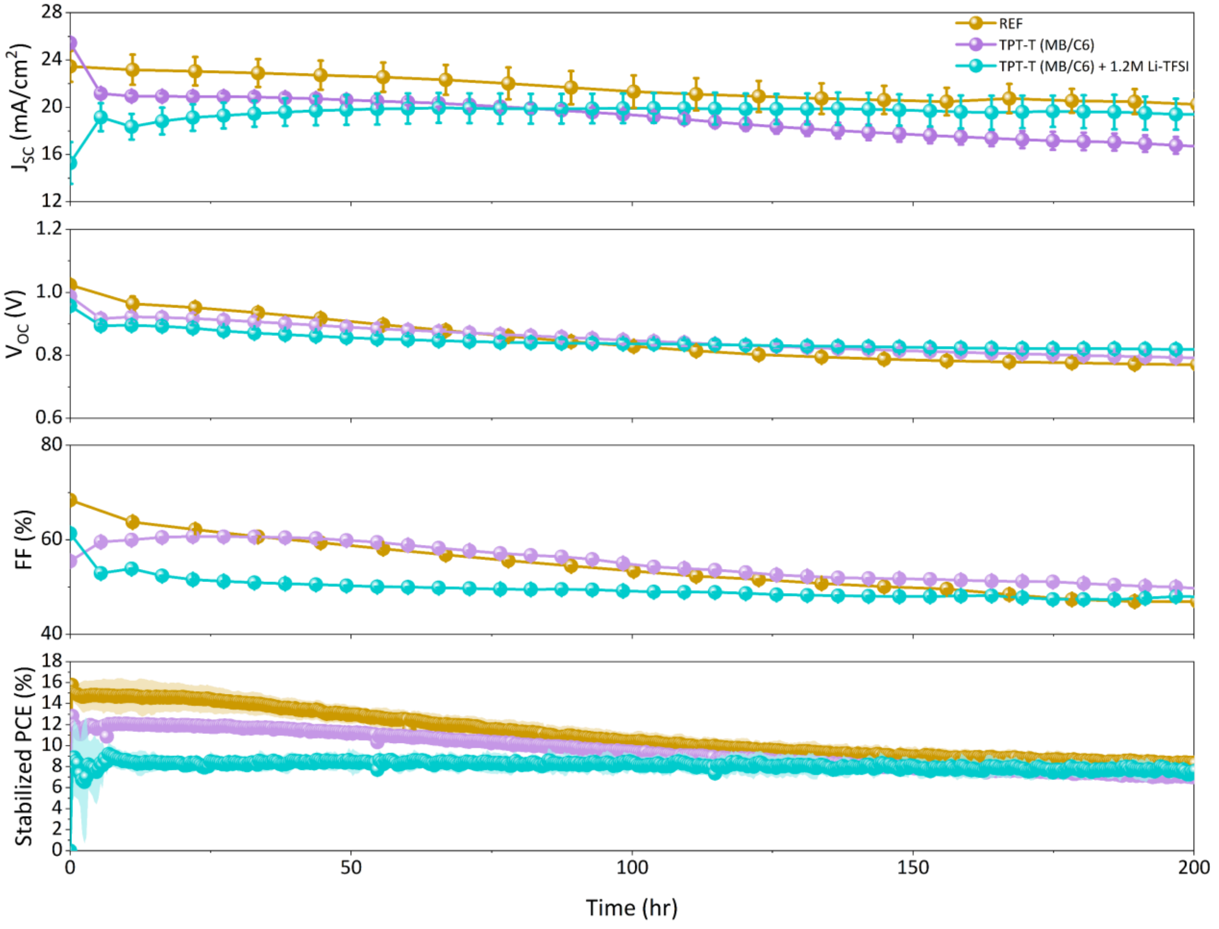

Figure S15. Evolution of the J-V parameters during long-term stability measurement of REF, TPT-T (MB/C6), and doped TPT-T (MB/C6). Photovoltaic parameters from J-V scans were automatically extracted every 12 hours during the MPPT at 65 °C. REF and 1.2M Li-TFSI refers to "doped Spiro-OMeTAD" used for control and combination of Li-TFSI and tBP additives, respectively.

References

(1) Sabury, S.; Xu, Z.; Saiev, S.; Davies, D.; Österholm, A. M.; Rinehart, J. M.; Mirhosseini, M.; Tong, B.; Kim, S.; Correa-Baena, J. P.; Coropceanu, V.; Jurchescu, O. D.; Brédas, J. L.; Diao, Y.; Reynolds, J. R. Non-Covalent Planarizing Interactions Yield Highly Ordered and Thermotropic Liquid Crystalline Conjugated Polymers. *Mater Horiz* **2024**, *11* (14), 3352–3363. https://doi.org/10.1039/D3MH01974H.

(2) Endres, J.; Egger, D. A.; Kulbak, M.; Kerner, R. A.; Zhao, L.; Silver, S. H.; Hodes, G.; Rand, B. P.; Cahen, D.; Kronik, L.; Kahn, A. Valence and Conduction Band Densities of States of Metal Halide Perovskites: A Combined Experimental–Theoretical Study. *J Phys Chem Lett* **2016**, *7* (14), 2722–2729. https://doi.org/10.1021/acs.jpclett.6b00946.

(3) Khenkin, M. V.; Katz, E. A.; Abate, A.; Bardizza, G.; Berry, J. J.; Brabec, C.; Brunetti, F.; Bulović, V.; Burlingame, Q.; Di Carlo, A.; Cheacharoen, R.; Cheng, Y. B.; Colsmann, A.; Cros, S.; Domanski, K.; Dusza, M.; Fell, C. J.; Forrest, S. R.; Galagan, Y.; Di Girolamo, D.; Grätzel, M.; Hagfeldt, A.; von Hauff, E.; Hoppe, H.; Kettle, J.; Köbler, H.; Leite, M. S.; Liu, S. (Frank); Loo, Y. L.; Luther, J. M.; Ma, C. Q.; Madsen, M.; Manceau, M.; Matheron, M.; McGehee, M.; Meitzner, R.; Nazeeruddin, M. K.; Nogueira, A. F.; Odabaşı, Ç.; Osherov, A.; Park, N. G.; Reese, M. O.; De Rossi, F.; Saliba, M.; Schubert, U. S.; Snaith, H. J.; Stranks, S. D.; Tress, W.; Troshin, P. A.; Turkovic, V.; Veenstra, S.; Visoly-Fisher, I.; Walsh, A.; Watson, T.; Xie, H.; Yıldırım, R.; Zakeeruddin, S. M.; Zhu, K.; Lira-Cantu, M. Consensus Statement for Stability Assessment and Reporting for Perovskite Photovoltaics Based on ISOS Procedures. *Nature Energy 2020 5:1* **2020**, *5* (1), 35–49. https://doi.org/10.1038/s41560-019-0529-5.
